# Examining the Pro-Self and Prosocial Components of a Calling Outlook: A Critical Review

**DOI:** 10.3390/bs13080684

**Published:** 2023-08-16

**Authors:** Rona Hart, Dan Hart

**Affiliations:** 1 School of Psychology, University of Sussex, Falmer, Brighton BN1 9RH, UK; 2 Birmingham Business School, University of Birmingham, 116 Edgbaston Park Road, Birmingham B15 2TY, UK; d.hart@bham.ac.uk

**Keywords:** calling, meaning of work, values, prosocial behaviors

## Abstract

Work on callings has burgeoned in the past 20 years, yet recent reviews exposed a lack of conceptual clarity and disagreements around its definition, components and measures. One lingering point of contention revolves around the element of prosociality: is a calling orientation primarily motivated by self-interest, prosocially orientated, or a mix of both? This conceptual paper reviews and examines the pro-self and prosocial component of a calling outlook, by examining and comparing the ways in which they feature in different calling subtypes: classic, neoclassic and modern callings. Our analysis suggests that these subtypes vary in where they are located on a pro-self–prosocial continuum: classic callings are located on the prosocial side of the axis, modern callings are located on pro-self side of the axis, and neoclassic callings can be situated in the middle of the continuum, integrating self-orientated and other-orientated motivations. Our analysis further suggests that these calling subtypes draw on divergent value systems: classic callings are propelled by self-transcendent values, modern callings are driven by self-actualization motivations, and neoclassic callings integrate both value systems. We therefore argue that the subjective experiences of pursuing a calling within each subtype pathway differ, although they may culminate in similar outcomes. The paper offers a novel framework for analyzing people’s calling that draws on their values.

## 1. Introduction

In the past decade, we have witnessed a surge in the literature on callings, both empirical and conceptual [1,2]. Despite the impressive development, several authors [1,2,3,4,5] detected significant scholarly gaps, and argued that research on callings still suffers from conceptual ambiguity around the definition of calling, its core components, and measures. A key point of confusion and contention is the prosocial aspect of a calling outlook [1,2,6], which raises an important question: should a calling orientation necessarily entail the pursuit of prosocial goals, or can it be driven by self-orientated goals? [6]. This point of discord is reflected in the lack of consensus around the overarching definition of a calling outlook, as well as around the identification and definitions of its subtypes [1,2].

In response to authors’ calls for conceptual clarity, we conducted an in-depth theoretical examination of the three subtypes of calling that are recurrently mentioned in the literature (see, for example, [7,8,9,10,11]) but not clearly defined or distinguished: classic, neoclassic and modern callings. Our aim is to advance the scholarship in this area by clarifying the common factors that bind these sub-constructs together into an overarching calling concept, as well as by making clearer distinctions between these calling subtypes to explain how they vary. Our main argument is that classic, modern and neoclassic callings differ in some of their core features, in particular their pro-self/prosocial facet, which reflects the different motivations, purposes, sense-making processes and experiences of those who manifest these outlooks.

The paper opens with a brief critical review of the research on calling, which highlights the areas of ambiguity around the different subtypes of callings, and their prosocial/pro-self component. The paper then goes on to offer an overview of the pro-self–prosocial continuum, by examining the literature on the key dimensions of these opposing orientations. This is followed by a brief explanation of our analytic approach. The following sections provide an in-depth analysis of the classic, neoclassic and modern conceptions of callings, delineating their key features and unpacking their pro-self/prosocial qualities. The last section compares and contrasts these concepts focusing on their pro-self/prosocial components and proposes a new theoretical framework for future research on callings to draw on.

## 2. Calling: A Critical Review

Perceiving work as a calling is widely regarded as a key feature of leading a meaningful life [1,2]. For those who hold this perception, the calling becomes a central driving force in their lives, and work is performed for the sense of fulfillment that it renders them, rather than for income or career progression [1,2,12]. Wrzesniewski and colleagues [12] first introduced the concept more than 25 years ago, through the work orientation model. Work orientation is an outlook that people assume towards their work which embodies their relationship with work: what purpose or function work serves for the person, what work means to them, and its significance.

Drawing on earlier literature [13], Wrzesniewski et al. [12] differentiated between three work orientations:

- Job orientation: People who perceive work as a job are mainly interested in the material gains that work can offer them, and do not expect work to meet their personal goals or interests. Thus, there is a degree of separation between work and other aspects of life, and there is no expectation for engagement or fulfilment at work.
- Career orientation: People who regard their work as a career are more invested in their work and see it as a part of their long-term career path and professional progress. They expect their employment to provide financial gains, as well as a route for progression within their profession and organization that will enable them to acquire seniority, power, influence or prestige.
- Calling orientation: People who view their work as calling see their work as meaningful for themselves and beneficial for society and thus an end in itself. They do not work for material gains, career advancement, prestige or power, but are driven by a strong intrinsic motivation and commitment to their work, fueled by the sense of significance and fulfilment that they experience when doing the work.

Importantly, Wrzesniewski et al. [12] take a constructivist approach in this model, which maintains that these outlooks reflect people’s dispositions and subjective sense-making constructions around their work. Therefore, these orientations do not solely stem from the objective features of the job, nor do they stem from the organizational environment. The authors found that employees who hold a similar position within the same organization may hold different work orientations, which justifies this approach. The authors’ key claim is that these perceptions significantly matter, since they shape people’s work-related behaviors, such as career goals, engagement with work, motivation and commitment, job satisfaction and work identity. Thus, the construct can provide a useful framework through which researchers can examine the impact that work-related meaning-making processes have on behaviors and outcomes.

Wrzesniewski and her colleagues [12] developed the concept further by creating a measure of work orientation and by conducting surveys across a variety of occupations to examine its manifestations. Their findings revealed that the three orientations can be found in most occupations. They also found that any work can be viewed as a calling. Based on correlational findings, they reported that those who perceive their work as a calling experience their work as profoundly meaningful, compared to other orientations. Each work orientation also predicted particular outcomes: respondents with a calling orientation showed significantly higher levels of wellbeing, greater job and life satisfaction, and less absenteeism compared to others.

Building on this primary work, scholars attended much more to the calling orientation compared to the other orientations, and the amount of publications on calling has soared [2]. In a recent meta-analytic review of the calling literature, Dobrow and her colleagues [2] found 625 papers published between 1997 and 2018. In another extensive review, Thompson and Bunderson [1] reported that the majority of papers focus on the outcomes of holding a calling orientation, with outcomes mainly analyzed from employees’ perspective. Fewer studies explored other aspects of calling, such as the antecedents that promote the development of a calling outlook, people’s experience of having and pursuing their calling, or the outcomes of holding a calling outlook for organizations.

In line with the current scholarly attention to the personal upshots of a calling outlook, in their meta-analytic review, Dobrow et al. [2] drew on 201 quantitative publications and addressed a central question: to what extent does a subjective perception of work as a calling promote or undermine key life and work-related outcomes (such as wellbeing, meaning in life, job satisfaction, engagement, commitment and performance)? They concluded that a calling orientation has “an extensive positive impact, even beyond our theorizing, on outcomes in both work and life” (p. 32) (see key findings below).

Despite the impressive progress made, two interlinked points of controversy have persisted in the literature. The first is the imprecision and lack of consensus around the overarching definition of a calling outlook. The source of the ambiguity in this meta-definition revolves around the confusing classification and definitions of several calling subtypes (classic, neoclassic and modern). These are frequently presented as a principal definition, rather than a subtype, hence resulting in multiple, incompatible definitions [6,7,11,14]. We maintain that this is because an overarching definition of a calling outlook that neatly encompasses these subtypes has never been offered. We briefly unpack these points below.

In reference to the debate around the overarching definition of a calling orientation, Thompson and Bunderson [1] (p. 428) made the following observation:

“The question of definition is clearly the elephant sitting awkwardly in the center of the room. Put simply, there is no clear and consensual definition of calling in the literature. As a result, researchers interested in the phenomenon of calling typically begin by acknowledging the diversity of definitions in the literature and then selecting one definition for their study, or proposing their own version …”.

Dik and Shimizu [4] maintained that these conceptual disagreements create numerous challenges for ongoing research. They have a knock-on effect on the development and use of measures, which tend to correspond with a particular model or definition, rather than encapsulating the overarching concept (see, for example, [7,10,12,14]). A similar critique was voiced by Vianello et al. [15], who argued that the multitude of measures used results in a lack of capacity to compare or interpret differences that emerge across studies, which hampers theory development.

We maintain that the key point of disagreement around the definition of a calling outlook revolves around its pro-self/prosocial element [2,4,6]: Should a calling orientation necessarily be driven by the pursuit of prosocial goals that benefit the greater good? Could it be driven by self-focused goals that promote self-fulfillment and self-realization? Or could they perhaps combine both self- and other-orientated motives and goals? Although researchers have touched on these points, they have not been thoroughly discussed or systematically analyzed, and therefore they remain blurred.

The second point of controversy and ambiguity is around the classification and definitions of three subtypes of a calling orientation: classic, neoclassic and modern. The debate on these subtypes seems to inform and feed into the dispute around its pro-self/prosocial element [1,2,3,4,5,6], as we shall demonstrate next.

In Wrzesniewski et al.’s [12] (p. 22) work orientation model, the authors incorporated into the calling outlook a prosocial component, and defined a calling orientation as a “meaningful beckoning toward activities that are morally, socially, and personally significant” [16] (p. 46). In line with this definition, Duffy et al. [17] maintained that callings entail the following key components: work contributes to the greater good; the person feels summoned to perform the work by an external source, and work generates a sense of meaning and purpose. Dik and Duffy [18] offered the following definition: “a transcendent summons, experienced as originating beyond the self, to approach a particular life role in a manner oriented toward demonstrating or deriving a sense of purpose or meaningfulness and that holds other-oriented values and goals as primary sources of motivation” (p. 427). It is noteworthy that “prosocial intention—a desire to make the world a better place” [9] (p. 429) is considered a core feature of this calling outlook, though there is an acknowledgment that it is not devoid of self-orientated intentions or gains, such as sense of purpose, personal significance and work meaningfulness. In the earlier literature [1,7,9,16], this perception was characterized as a “traditional” or “classic” calling orientation. However, in recent work, this orientation has been labelled as a “neoclassical” calling [1,2,4,17,19].

Over the years, however, other definitions of a calling outlook emerged that are situated closer to the self-focused side of the pro-self–prosocial axis. Dobrow and Tosti-Kharas [10] (p. 1003) defined a calling outlook as a “consuming, meaningful passion people experience toward a domain”. Adhering with this definition, Berg et al. [20] suggested that calling entails the following features: one feels intrinsically driven to pursue this line of work, the person experiences a good person–job fit, work is enjoyable and meaningful, and the person sees their occupation as part of his or her identity. Since this conception of calling does not entail a prosocial intent, Dobrow-Riza et al. [21] (p. 4) suggested that researchers “need to loosen the assumption that callings “must” be other-oriented.” This conception is labelled in the literature as a “modern” calling [1,2,4,10,21].

A third definition that integrates prosocial and pro-self-interest intents and behaviors, and which is therefore located in the middle of the self-orientated–other-orientated continuum, was offered by Elangovan et al. [9] (p. 430): “a course of action in pursuit of prosocial intentions embodying the convergence of an individual’s sense of what he or she would like to do, should do, and actually does.” Taking a similar line of reasoning, Thompson and Bunderson [1] (p. 432) defined a calling outlook as “a conviction—often felt as a sense of destiny or fit that a particular domain of work leverages one’s particular gifts and consuming passions in service of a cause or purpose beyond self-interest”. This orientation is mostly labelled in the literature as a “neoclassical” model of calling [4,7,19], as Dik and Shimizu [4] (p. 325) explained: “neoclassical callings share the core element of calling as a meaningful and purposeful approach to work but tend to preserve the historic notion that a calling is motivated by a prosocial desire to use one’s gifts toward positive societal impact”. We note, however, that in a recent review, Thompson and Bunderson [1] labeled this calling outlook as a “transcendent calling” (p. 421).

As demonstrated above, although researchers seem to acknowledge the existence of calling subtypes [1,2,4,7,9], each of the descriptions cited above was presented as a primary definition of a calling outlook, resulting in several discordant characterizations [6,7,11,14,16]. For example, Thompson and Bunderson [1] (p. 432) maintained that their definition (cited above) of the “transcendent calling” subtype should be used as an overarching definition of a calling outlook, since it “may promise a solution to the definitional stalemate in the calling literature”. However, we argue that similar to earlier work, this definition has been proposed since it corresponds with the authors’ distinctive subtype model of calling, therefore excluding other models and conceptions. Unfortunately, such turf wars have been endemic within the calling literature, and we suggest that they may be the cause of the lingering lack of agreement around the overarching definition of the concept.

Despite the apparent prominence of the pro-self–prosocial continuum in the overarching definition of a calling orientation, and in the classification of its subtypes, to our knowledge, no earlier paper explicitly explored the concept of pro-self/prosociality with the purpose of examining their connection with the calling construct. Earlier work did, however, offer some indications of this line of reasoning. For example, Thompson and Bunderson [1] differentiated between three calling subtypes: One type is driven by “inner requiredness”—a person’s needs, desires, interest and quest for self-realization. A second subtype is responsive to and driven by “external requiredness”. It is a socially orientated calling outlook, motivated by a sense of duty and civic responsibility. As for the third calling subtype, the authors claimed that the most powerful experience of a calling occurs when both internal and external requiredness are present and interact. As noted, the authors characterized this calling outlook as “transcendent calling”.

Similarly, Dobrow and her colleagues [2] differentiated between two primary low-level calling paths: an internally driven, self-focused calling, and an externally driven, other-focused calling. They also highlighted the existence of a “higher-order calling factor that is composed of two correlated yet distinct lower-order calling types” (p. 26).

An intriguing point to highlight is that Dobrow et al. [2] reported that these calling subtypes seem to culminate in similar outcomes (career self-efficacy, decision making, work engagement) and differ (though minutely) in the following ways: other-focused calling were more strongly associated with meaningful work and with eudemonic wellbeing, while self-focused callings were more strongly associated with job satisfaction and hedonic wellbeing. Hence, the authors argued that these findings challenge the current thinking that these calling subtypes are distinct:

“Our evidence highlights how strongly the two types relate to each other. Internally and externally focused callings not only lead to quite convergent outcomes but are also substantially correlated”.

[2] (p. 29)

It is noteworthy that in both papers [1,2], the authors differentiated between three calling subtypes, one of which is other orientated, a second subtype is self-orientated, and a third subtype that integrates pro-self and prosocial intents, a key point that we further unpack in the current paper. Intriguingly, both authors chose to use terms such as inner/outer requiredness and internally/externally driven calling outlooks, which lack theoretical grounding or empirical work, rather than referring to prosociality/pro-self concepts—two domains with a rich history and abundance of research and conceptual work (see review below). Another point to highlight is that Dobrow et al. [2] claimed that due to culminating in similar outcomes, the calling subtypes are indistinct. We challenge this assertion, and argue that since these findings draw on statistical (mainly correlational) analyses, the similarities found between outcomes may be caused by the use of calling questionnaires that have several overlapping components. Hence, the observed convergence reflects both the weak construct validity of questionnaires that measure each subtype and the high convergence validity that exists between these scales.

Drawing on these points, in this conceptual and critical paper, we aim to address this gap in the literature by theoretically “connecting the dots” between the calling construct and the concepts of pro-self/prosociality, to consider how prosociality or pro-self orientations play out within each of the calling subtypes. For this purpose, we first offer a brief overview of the concepts of prosociality/pro-self orientation.

## 3. Prosocial and Pro-Self Orientations: A Brief Introduction

### 3.1. Prosociality

Research on prosociality has a long history spanning more than four decades [22]. Although it originated in psychology, it is currently emerging as a multidisciplinary scholarly field, advanced by scholars from a wide range of disciplines, including education, business, social work, health care and more [22]. Within psychology, prosociality is conceptualized as a multidimensional umbrella term that includes an array of voluntary behaviors, dispositions (traits, states, perceptions, intentions or motivations) or processes that benefit others or focus on the welfare of others [23]. An earlier definition [24] considered that prosocial behaviors contribute to the wellbeing of others—a person or a group valued by society, aligned with its norms and incurs positive social consequences.

According to Penner et al. [25], prosocial behaviors, dispositions and processes can occur at three levels:

- The micro level focuses on intraindividual factors and examines prosocial dispositions, tendencies or motivations that are other-orientated and intended to benefit them. This level contains concepts such as prosocial personality [26], agreeableness [27], empathy and sympathy [28], compassion [29] and perspective taking [30].
- The meso level is interpersonal and explores behaviors or actions that aim to benefit a person or a small group. This level includes behaviors such as helping [31], acts of kindness [32], altruism [33], caring [34], social support [35], prosocial spending [36], generosity [37], Organizational Citizenship Behaviors [38] and heroism [39].
- The macro level focuses on prosocial behaviors or processes that occur in larger contexts such as groups, communities or organizations. This category includes prosocial behaviors enacted by an individual, group or organization which target a large entity such as an organization, a community or a wider social cause. It therefore includes behaviors such as donating [40], volunteering [41], social activism [42], servant leadership [43] and social entrepreneurship [44]. At times, they can manifest as a norm, a process or a set of values within a society or an organization, such as corporate philanthropy [45], Public Service Motivation [46], Corporate Social Responsibility [47] or Environmental, Social and Corporate Governance [48].

An important point relating to Penner et al.’s [25] multilevel classification is that dispositions are seen as a key aspect of prosociality whether they manifest in a behavior or not. According to Pfattheicher et al. [49], the mere focus on others with a genuine intent to promote their welfare satisfies the criteria for a disposition to be considered prosocial, regardless of whether it leads to action, and irrespective of the consequences, which may be ineffective and/or at times even detrimental.

Another central point relates to the motives that underlie prosocial behaviors: Although prosocial behaviors are intended to benefit others, they do not exclude the possibility that they may benefit the giver as well as the receiver. Furthermore, there is an acknowledgement in the literature that prosocial behaviors are not always driven by prosocial intents or motivations, and that they can be driven by pro-self, egotistic intentions and goals [50]. Consequently, in much of the research on prosocial behaviors (particularly in organizational settings) there is a recognition that prosocial behaviors should be evaluated through their outcomes, rather than through their intents, therefore leading researchers to focus on the outcomes of prosocial behaviors [49,51].

A third point that is essential to consider is the voluntary/non-voluntary nature of prosocial behaviors. The term ‘voluntary’ in the definition offered above suggests that in order for a behavior that is other-orientated to be considered prosocial, it needs to be self-initiated by the actor, as opposed to behaviors that are role prescribed, which are conducted as part of a person’s job [52]. However, this nuanced distinction has not been applied within much of the management literature. For example, Bolino and Grant [51] (p. 602) defined prosocial behaviors as “acts that promote or protect the welfare of individuals, groups, or organizations” and noted that these behaviors can be role prescribed (in-role behaviors) or discretionary (extra-role behaviors). As this study refers to the work domain, we adopt here the wider concept as it is applied in the business literature, though we will highlight the voluntary/non-voluntary nature of prosociality in the analyses that follow.

There are numerous factors that influence a person’s inclination to engage with prosocial behaviors. These include demographic features such as gender [53], age, religiosity and ethnicity [54]; personality traits such as prosocial personality [26], empathy [28], perspective taking [30] and agreeableness [27]; emotional states such as positive emotions [55] and compassion [29]; attitudes such as a social responsibility mindset and just world belief [26]; as well as contextual and situational factors such as cultural norms [56], cost–benefit analysis [57] and situational attributions [52]. Since these are rarely mentioned in the calling literature, we have not provided a full review of the literature here.

### 3.2. Pro-Self Orientation

In psychology, the opposite side of prosocial dispositions and behaviors are often considered selfish, ego-centered, self-serving and even antisocial outlooks and actions [58]. However, within the calling literature, the pro-self intentions and actions categorically do not fit with these descriptions, and they are more aligned with ideas around a healthy, agentic, self-determined and autonomous pursuit of self-interests [2,10].

Self-interest refers to the inclination to act in ways that primarily focuses on the satisfaction of one’s needs, desires and interests and on bringing potential benefit to oneself [59,60]. These could include achieving personal goals, protecting personal rights or pursuing personal wellbeing and satisfaction. Self-interest is linked to the concept of agency, defined as an individual’s ability to act independently and make free choices [61]. It is through the exercise of agency that individuals can pursue their self-interests.

Baumeister [62] argued that self-interest is a natural human motivation, and when it is adaptive, it involves a balance between concern for oneself and concern for others. Therefore, self-interest goals or motives are not necessarily in conflict with other people’s interests or with societal objectives. The economist Adam Smith [63] maintained that self-interest can lead to positive outcomes for societies, since individuals who pursue their own self-interest contribute to a well-functioning society: “it is not from the benevolence of the butcher, the brewer, or the baker that we expect our dinner, but from their regard to their own self-interest” [63] (p. 13). While goals rooted in self-interest can be externally driven, like seeking wealth or fame, they can also be internally motivated, such as striving for authenticity, self-expression, pursuing personal interests, achieving individual goals or living in alignment with one’s values.

Self-interest can be displayed through varied dispositions, such as a sense of individualism and independence (as opposed to interdependence) [64], which can manifest in perceiving oneself as “separate from others, autonomous from the world and relatively unique” [65] (p. 141). It is also linked to achievement motivation [66], self-efficacy [61] and a belief in personal uniqueness [66].

Self-interest can manifest in a variety of behaviors, such as pursuing individual goals, acting independently of others, considering or prioritizing one’s own needs, interests or desires in decision making and when taking action, and pursuing personal gains [65]. It is also linked to self-preservation behaviors that are geared to protect one’s health, wellbeing, assets or status quo [67]. Defensive or protective behaviors are also associated with self-interest and can include risk avoidance and protecting oneself from physical, emotional or reputational harm [68]. Another feature associated with self-interest is resource accumulation, which could be financial, informational or related to other assets [69].

In their relationship with others, those with a strong self-interest are inclined to seek self-validation, recognition, reward, control, power or other means of self-enhancement or self-promotion [66], and are more likely to engage in social comparison and competitive behaviors to gain resources, recognition or power [70,71]. Interestingly, self-interest was found to be associated with hedonism: seeking pleasurable experiences, immediate gratification and transitory satisfaction [65]. On the negative side, self-interest may lead to lack of consideration to other people’s needs or views [72], competitive behaviors, and lack of cooperation (such as withholding information, support or resources from others). Notably, context matters: cultural factors such as individualism can promote self-interest by emphasizing the importance of personal achievement and success [64].

Another interesting distinction made by Cohen et al. [73] suggests that people’s self-perception differs along the pro-self–prosocial continuum. The authors differentiated between holding an insider and outsider perspective, and found that people with high self-interest tend to hold an insider perspective. It is manifested in the tendency to dwell in their own private, internal experiences, which may result in projecting their experiences onto others or to life events. In contrast, those with an outsider perspective tend to see themselves from others’ perspective. The authors also found that self-interest is linked to the presence of several cognitive biases: egocentricity—seeing the self as the focus of attention; beneffectance—assuming responsibility only for positive but not negative outcomes; and cognitive conservatism—resistance to change [73].

It is beyond the scope of this paper to fully review the vast literature on prosociality and on self-interest and to explore the varied forms in which they appear, their antecedents and consequences. Rather, our purpose in this section is to offer a foundation for the analysis that follows on the role that prosociality and pro-self orientations play within the calling outlook.

## 4. The Current Paper: Calling and Prosociality/Pro-Self Orientations

Earlier, we demonstrated that in the definitions of the calling subtypes, the pro-self–prosocial continuum notably plays a primary factor in distinguishing between them. However, it is unclear how these prosocial/pro-self orientations manifest in their core components and measures, and in their outcomes, since these have not been thoroughly explored in earlier work. In this paper, we address this void in the literature by offering a conceptual analysis of each calling subtype, looking specifically at the pro-self/prosocial aspects of each of the calling subtypes. The analysis draws on both empirical and theoretical literature to examine the following dimensions:

- The definition and components of calling: To assess how the dimensions of pro-self/prosociality feature in each calling subtypes, we review the definitions and key components of each calling subtype.
- The measures of calling: To examine the ways in which the calling subtypes are measured, and which dimensions of pro-self/prosociality are included in these scales, we review and discuss the prominent measures of calling that match each subtype.
- The pro-self–prosocial continuum: To investigate how the pro-self/prosocial dispositions and behaviors manifest in each of the calling subtypes, we examine and explain their positioning along the pro-self–prosocial continuum. We also review the literature to create a clearer picture of what types of pro-self or prosocial behaviors, dispositions or processes may be involved in each calling subtype.
- The outcomes of pursuing a calling: The analysis presented below reviews the personal and work outcomes of each calling outlook as reported in empirical research, including the “dark side of calling”—the personal costs that individuals experience as they pursue their calling [74]. Particular attention is paid to outcomes that revolve around or linked to the pro-self/prosocial aspect of each calling subtype.

## 5. Analytic Approach

In the conceptual analysis that follows, we present a brief review and analysis of classic, modern and neoclassical calling subtypes, focusing on their pro-self/prosocial components.

We focus on the experience of living a calling as opposed to having a calling (but not pursuing it), a distinction made by Duffy and his colleagues [75], since we consider that the differences between the calling subtypes are more relevant and distinct when one is pursuing his or her calling through a particular vocational route. This is because differences in dimensions such as prosocial intent and behaviors, work meaningfulness and identification with work are less likely to manifest when people hold a calling outlook but are not living it.

To unpack, compare and contrast the components of classic, modern and neo-classical callings, and the ways in which their position on the pro-self–prosocial continuum manifest in each subtype, we conducted a search of the theoretical and empirical calling literature, specifically looking for empirical papers that mention the terms classic, secular, religious, spiritual, modern or neo-classical calling, for theoretical or empirical papers that discuss or make a distinction between subtypes of callings (see, for example, [1,2,4]). From this body of research, we elicited the following three components:

- The definition of the calling subtypes offered by the authors.
- The core components of the construct.
- The measures of the construct.

This enabled us to map the three types of calling and outline the key differences between them, as well as match each type of calling with its corresponding empirical measures.

We then conducted a broader literature search, specifically looking for papers that draw on these measures to further unpack the ways in which the pro-self/prosociality dimensions are manifested in each subtype of calling, and we extracted the data on the points noted above. Hence, the analysis that follows is mostly drawn from quantitative papers and theoretical work. In cases where we were able to identify qualitative papers that adhere to a particular calling subtype, we added these to the analysis.

We note that the following scales were not included in the analysis: the Brief Calling Scale [14], the Living One’s Calling Scale [76], the Calling Motivation Scale [77] and the Vocational Identity Questionnaire [78] were excluded due to not enabling a classification as to which subtype the calling orientation adheres to. Scales not available in English (such as The Chinese calling scale, Chinese Calling Scale (CCS) [79], were also excluded. The Career Calling Scale for Emerging Adults (CCS) [80] was removed due to its focus on emerging adults who are not yet living their calling. The Work Orientation Scale [12] was excluded due to encompassing a larger construct that includes other orientations (job, career) in addition to calling.

An additional point to make is that this review does not claim to be a complete and exhaustive review of the literature on this subject, since that is not within its remit. Rather we sought to find sufficient evidence in the literature to facilitate a rigorous examination of the relevant literature and to answer the research question with confidence.

## 6. The Classic Calling Orientation

The classic calling orientation has two subtypes: a classic religious orientation and a classic-secular orientation.

### 6.1. The Classic Religious Calling Subtype

Early conceptions of calling were rooted in several religious philosophies [9,16,81,82]. The bible includes several narratives of people who were called by God to conduct sacred work. This often meant embarking on a prosocial life-long vocation in the public service [9,82] and leading a lifestyle that adhered to a particular moral code that often required self-sacrifice or hardship [83,84]. People who pursued a religious calling often perceived it as their moral duty to serve the greater good, as well as their destiny, as they were acting on God’s plan for their lives [9,82].

Christopherson [83] (p. 219) defined a religious calling (often used as a synonymous with spiritual calling) as “a task set by God with a sense of obligation to work for purposes other than one’s own”, maintaining that it entails the following key components:

- One feels summoned by God to perform the work;
- Work is prosocial;
- A sense of duty or stewardship;
- An alignment of the work with one’s life purpose;
- A connection between one’s religious identity and work;
- Work is deeply meaningful.

These components are captured by The Faith at Work scale, developed by Lynn et al. [85]. The questionnaire includes 15 items and covers the following dimensions of the religious calling construct: a sense of being called, a transcendent summon from a divine source, a sense of moral duty, an alignment of one’s religious identity with the work, being pre-destined to perform the work, work is prosocial, a sense that work is one’s life purpose and deeply meaningful, and willingness to sacrifice. Examples of items include the following: I view my work as a mission from God (calling); I view my work as part of God’s plan to care for the needs of people (prosocial/purpose); I think of my work as having eternal significance (meaning); I view myself as a caretaker not an owner of my money, time and resources (stewardship). The scale does not include items that measure person–job fit or enjoyment.

It is noteworthy that in several recent reviews [1,2,4], classic callings as a category (and particularly its religious subtype) has been omitted from the review, suggesting that it has withered, as it is less relevant to today’s world of work. However, Thompson and Bunderson [1] acknowledged that religious callings (and research that explored them), although rare, still exists in contemporary employment, particularly in religion-related occupations such as ministry [83,86,87,88,89], staff employed by religious universities [90,91], religious teaching [92] and leaders of religious charities [93,94].

Religious callings are firmly positioned on the prosocial side of the pro-self–prosocial continuum, featuring other-orientated dispositions or behaviors. This is due to serving God’s will and the greater good, and expressing a sense of stewardship, civic duty or social responsibility [83,85,91].

Prosociality can emerge in this model in a variety of forms, and although they may encompass all levels in Penner et al.’s [25] classification (micro, meso and macro), most of the literature documents prosocial dispositions and behaviors that occur on the micro (intrapersonal) or on the meso (interpersonal) levels, and there is scarce literature on the macro level.

Examples of prosocial dispositions mentioned in the literature include the following: a capacity to value, accept, empathize and care for others [83,88], openness to others, awareness of others’ needs and concern for them [83], a broader sense of “transpersonal responsibility” or a “concern for broader societal and social justice issues” [86] (p. 140).

Prosocial behaviors mentioned in the literature include helping others, being present, comforting, supporting, listening, sharing, consulting, educating, volunteering and mentoring [83,86,91], and at times displaying a broader notion of “making a difference to people’s lives” [94] (p. 411). In a few studies, they took the form of more complex behaviors such as servant leadership and social entrepreneurship [83,93]. However, it is difficult to ascertain from the available literature whether these behaviors are voluntary or role prescribed, a point that requires further inquiry.

Christopherson [83] maintained that selflessness is a key feature of religious calling. Selflessness can manifest in this model through several aspects.

- Service orientation: Work that is perceived as a spiritual calling is often described as a service-focused endeavor whereby one’s labor is seen as a service to God, as well as to society, and it therefore requires a person to prioritize God’s directives and others’ needs, above their own [83,84,90,91].
- Duty and responsibility: Selflessness also comes into play through the perception of work as one’s “sacred responsibility” [92] (p. 101) or social obligation [3,83,87], experienced as one’s civic duty [95].
- Destiny: Several authors reported that people who view their work as a spiritual calling often feel predestined to perform the work [83,84] due to being summoned by God, and doing what they were “meant to do” [93] (p. 95). Weber (cited in Goldman) [96] (p. 110) noted that “the calling is not primarily a source of self-satisfaction or the satisfaction of craftsmanly desires, nor is it seen as the fulfilment of talents or of satisfying involvement with an activity that they love. Instead, it serves the needs of self-definition, self-justification, and identity through devotion to a higher ideal throughs service”.

It is worth noting that the transcendent summon to perform the work is entwined with these points—the service orientation, sense of duty and destiny—and seems to underlie and provide the justification for these prosocial and selfless dispositions and behaviors. Due to the sense of being summoned by a divine source to perform the work, the “calling is framed as what one ‘ought to’ do rather than what one chooses to do” [3] (p. 170), and those who perceive their work as a religious calling, often describe a compelling need to follow God’s call, which draws them into an occupational path that is not always of their own choosing [3] and where they become “a vessel suitable for godly work” [83] (p. 234).

Within the literature on prosociality, selflessness is a key component only in one concept: altruism. It is defined as dispositions or behaviors that are intended to benefit others and that are characterized by selfless intentions, with no expectations of reward or benefit, that may incur a cost to the person who performs it [58].

The pursuit of a religious calling is therefore other-orientated, and can be seen as self-transcendent [3,97]: “being a part of something bigger” [90] (p. 424). Hartman and Zimberoff [98] defined self-transcendence as serving a purpose greater than the self, with a prosocial intent. It involves an expansion of a person’s consciousness, beyond their personal, self-centered ego and needs, to include other people and social concerns within that expanded identity [99]. In Schwartz’s [100] values model, ‘self-transcendence’ and ‘self-enhancement’ are two sides of the same axis. While self-transcendence involves the values of universalism and benevolence, self-enhancement is seen as a contrasting set of values, entailing power and achievement. Frankl [101] and Wong [102] argued that self-transcendence is a key factor in finding meaning and fulfilment in life.

Beyond the prosocial aspects that are associated with holding this calling outlook, several personal and work outcomes have been mentioned in the literature. On the personal side, religious calling has been linked to a person’s identity [83,103], in particular their religious identity [97,104,105], church attendance [104], faith maturity [104] and self-awareness [84]. It is also associated with undergoing personal development and growth [83], often experienced as a spiritual journey [84]. Several authors [85,90] reported on seeing work as one’s life purpose: a “sacred lifework… that gives meaning, purpose, and direction to life” [91] (p. 98), and others found an association between spiritual calling and meaning in life [85,106].

Work-related outcomes reported in the literature include a positive association between the calling outlook and job satisfaction [88,94,106], regardless of fit [106]. Neubert and Halbesleben [106] found that the calling instils work with meaning, purpose and direction. Greene and Robins [94] reported that clergy experienced autonomy in their work. Religious calling was also positively associated with organizational loyalty [103] and organizational commitment [88,90,107], whether or not the job itself is fulfilling or satisfying [106]. However, in one study [107], the calling outlook was negatively correlated with performance, and positively associated with intents to leave.

Religious callings are also associated with costs and sacrifices, and these seem to be linked to the prosocial aspect of the work. Neubert and Halbesleben [106] noted that it can drive a person to stay in a job that lacks fit with their skills or is a negative environment, due to their sense of duty. Several authors [90,93,95] reported on overwork among those with a spiritual calling, and a tendency to sacrifice their time, income, resources, and personal and family lives for work. Dunbar et al. [108] found high burnout levels in Christian ministry, which has been attributed to several causes: being called during stressful emergencies, vicarious traumatization, emotional labor, working outside of work hours, work–family infringement, inter-role conflict and physical/psychological impairment. Similarly, Lee [109] reported on high demand emanating from congregation members, resulting in pastors sacrificing their personal and family lives to support others, which can lead to work–family conflict, identified as one of the key causes of pastoral burnout.

On a societal level, Weber [110] argued that this calling orientation is associated with a hierarchical outlook of the realm of employment, which characterized early Christian theology. This is because people whose callings emerged from their relationship with God often perceived themselves, and were seen by others, as the “chosen few” whose work was a calling, and therefore, they had a superior social status, in contrast to the menial work that the majority of people performed [81]. This resulted in sharp social divisions between occupations that stemmed from a judgement about their social worth. This conception prompted to the emergence of a secular classic calling orientation described next.

### 6.2. The Classic Secular Calling Subtype

The Protestant Reformation introduced a significant change in how callings were perceived. Drawing on Luther‘s [111] teachings, the reformers endorsed the idea that all types of work are callings. They also claimed that by conducting their work faithfully and productively, workers serve a higher purpose [111]. Calvin [112] expanded on Luther’s work by suggesting that callings are uniquely personal. He asserted that when people use their God-given talents to benefit the greater good, they fulfil their callings [81,84,113].

Post-reformation calling ideas proposed that due to interdependence, people have a duty to contribute to their communities by realizing their callings [11,113]. Failure to do so was perceived as wasting one’s God-given gifts and not fulfilling one’s civic obligations and, therefore, morally wrong [3].

Weber [110] argued that these conceptions that all types of work can be seen as callings laid the foundation for the Protestant work ethic. Later, these ideas also led to the development of modern capitalism, since they offered the ideological rationalization for the obedient acceptance of one’s place in the modern employment hierarchy. Several authors noted that these developments led to the weakening of the religious component of calling, particularly the notion that people are called by God to conduct holy work [9,11,16,114].

In the decades since the Reformation, the idea of work as a calling has become secularized and spread globally, as an orientation that embodies the connection between a person and his or her work. Prosciality remains central in the secular perception of calling, which suggests that a calling entails work that is morally and socially worth doing, which contributes to other people’s welfare, promotes a worthy social cause or benefits the greater good more generally [8].

Duffy et al. [17] (p. 426) defined a secular calling as “an approach to work that reflects seeking a sense of overall purpose and meaning and is used to help others or contribute to the common good”. Duffy et al. [115] observed that in classic secular calling, people perceive their calling as a call to do a specific work that emerges from a secular source, such as an organization, community, family legacy, government, nation or a social mission. It should be noted, however, that in much of the empirical work on this category of calling, the source of the calling is not clearly identified.

Dik and Duffy [18] offered the following core features of classic secular callings:

- One feels called to perform the work by an external source (community, organization, others’ needs);
- Work is prosocial;
- Work aligns with one’s life purpose;
- Work is deeply meaningful.

These components are captured by the Calling and Vocation Questionnaire developed by Dik et al. [14]. The scale includes 24 items and has three subscales: transcendent summons—being called by an external source, purpose—work is purposeful and meaningful, and a prosocial orientation. It also incorporates two vertical subscales: presence—denoting that a person is living their calling, and search—when a person is searching for their calling. Items include the following: I believe that I have been called to my current line of work; my work helps me live out my life’s purpose; my work contributes to the common good; the most important aspect of my career is its role in helping to meet the needs of others; making a difference for others is the primary motivation in my career; my career is an important part of my life’s meaning. This calling measure does not entail a sense of duty, a sense of destiny, a person–job fit or work enjoyment.

Despite the argument in several recent reviews that classic secular callings are disappearing [1,2,4], other reviews [8,9,16,116] suggested that secular callings can be found in contemporary employment, particularly in occupations that are prosocially orientated such as leadership [117], teaching [118,119], healthcare and medicine [120], academic faculty [121], managers [122], IT professionals [123], train drivers [124], environmental activists [125] and volunteers [126].

Duffy et al. [17] (p. 425) maintained that “this approach to conceptualizing calling aligns more closely with the historic usage of the term [113], while broadening its application to a wider population than explicitly religious classical definitions”.

This perception aligns with Thompson and Bunderson’s [1] and Dobrow et al.’s [2] classification presented earlier, which made a distinction between three calling subtypes, one of which is driven by external requiredness (or externally driven), and is other focused, which seems to be closely aligned with the classic secular subtype.

Similar to religious callings, classic secular callings are firmly situated at the prosocial side of the pro-self–prosocial axis, due to its emphasis on motives and behaviors that benefits others [14]. In some papers this is accompanied by a sense of duty or social responsibility [120,125,126,127].

In this model, prosociality can emerge in varied ways, and can include all levels of prosociality (micro, meso, macro) in Penner et al.’s classification [25], though in the literature, much of the work focuses on the micro and meso levels.

At the micro level, intrapersonal prosocial dispositions documented in the literature include empathy [128], deep connection to other human beings [125], caring [118], a prosocial or altruistic motivation [14,117,127,129] a desire to benefit others [14,121,128], a “compassion to helping humanity” [120] (p. e3), a desire to “make a difference” [125] (p. 48) or to “contribute to other people’s lives” [128] (p. 201). These motives and attitudes are deeply rooted in other-oriented values [8] or, at times, to a strong emotional or spiritual connection to humanity [125].

Another repeated theme in the literature is a “sense of ethical responsibility” [127] (p. 15) or civic duty [120,125,126], a service orientation [125] or stewardship [117]. These are manifested in self-transcendent life goals (community feeling, spirituality, and conformity goals) [130], “a goal of making the world a better place” [118] (p. 31), seen as “expressing a higher purpose” [131] (p. 48).

On the meso level, interpersonal prosocial behaviors mentioned in the literature include “helping others, doing good, being needed” [120] (p. e3), organizational citizenship behaviors [132,133], making a meaningful contributions to one’s community [134], altruistic behaviors [117] and leading “a life of service” [120] (p. e3) directed at a particular client group or community [131]. However, it is difficult to establish from the literature whether these behaviors are voluntary or non-voluntary, and this point merits further inquiry.

Resembling the religious calling orientation, these dispositions or behaviors seem to be grounded in and emerging from the underlying sense of being summoned to conduct the work. In some papers, this is described as a force beyond one’s conscious awareness that invites the person to embark on a particular occupational path, “to do that which we were intended to do” [125]. (p. 109), hence denoting that, in some cases, a sense of destiny is also experienced [126,134].

Akin to the religious calling subtype, this view of calling coincides with the concept of self-transcendence. Frankl [101] argued that in order to find meaning in life, people are required to shift their focus away from their own needs and desires, and find ways to contribute to others. Lepisto and Pratt [5] used the term “justification perspective” for this calling orientation, which revolves around the pursuit of a worthy social cause, as they are highly legitimized and socially valued.

In addition to the prosocial elements noted above, other outcomes of holding this orientation have been widely studied. Secular calling was positively associated with life satisfaction [76,130,135,136], happiness [134], joy [134], improved health and health satisfaction [12], life meaning [76,134,135,137], character strengths usage [130], wisdom [117] and religiosity and spirituality [130,138].

In the work domain, secular calling was found to be positively correlated with work engagement [134,139], study, job or career satisfaction [76,121,135,136,137,140], passion [134], work enjoyment [141], work hope [14,137], a sense of purpose at work [132,139,142] or work direction [119]. Dik et al. [14] reported on a positive association between a secular calling outlook and intrinsic as well as extrinsic work motivation. It is also correlated with performance [119,122,123,124], putting extra efforts into work [117], effectiveness [117], productivity, investment and willingness to handle work challenges [119,121], devotion, service ethic, and lower absenteeism [133]. People with a secular calling orientation also show an identification with the work or with the organization [99,143], organizational and career commitment [119,140], work resilience and constancy despite a turbulent occupational environment [141] and low turnover intentions [140,143].

Other authors reported on positive association between calling and career self-efficacy [130,137] or career decision self-efficacy [14], career adaptability, career confidence, control and concern [132] and career outcome expectations [142]. Several studies found that calling was negatively correlated with job stress [144] and with burnout [119], though as we note below, there are some contradictory findings around these points.

In exploring the dark side of this calling subtype, several key points have emerged. Secular calling was associated with making personal sacrifices for work [134]. Allan and Duffy [130] reported on a negative association between the calling outlook and financial success, hedonism, safety, and physical health goals. Other studies reported on personal needs not being a priority [125] and therefore being overlooked. Johansson and Hamberg [120] reported on medical doctors seeing their work as a “lifelong enterprise implying personal sacrifices” (p. e3) and experiencing “highly demanding duties, such as heavy responsibilities, long working hours, many nights on duty, readiness for service, and a never-ending learning commitment” (p. e3). Several studies reported on overwork [125], inability to disconnect from work [125,134], “feeling overwhelmed, discouraged, and depressed” [125] (p. 112), emotional exhaustion [119], “feeling like there was no more to give” [125] (p. 111) and burnout [125]. Hence, akin to religious callings, pursuing a secular callings has significant personal costs and sacrifices, and these seem to be linked to the prosocial aspect of the work.

Earlier we noted that in prior work, this outlook has been referred to as “classic” or “secular” calling [1,7,9,16], while in recent work, this orientation has been labelled as a “neoclassical” calling [2,4,17,19]. Although the change in title has not been clearly explained, we suggest that this is may be because there is an acknowledgment that despite the centrality of prosocial intents and behaviors in this model, it is not devoid of self-orientated gains, as seen in the numerous positive individual outcomes reviewed above. Nevertheless, we maintain that since an internal source of the call, and other items that demonstrate a pro-self orientation (see section below), are not captured by the available measure, we placed this subtype on the prosocial side of the pro-self–prosocial axis, and for this reason, we argue that the term classic-secular is more fitting for this calling outlook.

Our analysis suggests that religious and secular callings share the majority of their key features, and differ in two ways: The source of the calling in religious callings is divine, while in secular calling it is a community, a profession, a family legacy or an organization. Additionally, while both calling orientations are prosocial, there is evidence to suggest that one of the key characteristics of a religious calling is altruism (selflessness), which does not feature in the secular calling subtype.

## 7. The Modern Perspective of Calling

In a study on the calling orientation of musicians, artists and businesspeople, Dobrow and Tosti-Kharas [10] defined modern calling as a “consuming, meaningful passion people experience toward a domain” [10] (p. 1005) and identified several key features of this calling subtype:

- The call to conduct the work emerges from within;
- Work is linked with one’s vocational identity;
- Work aligns with one’s life purpose;
- One experiences good person–job fit;
- Work is intensely meaningful;
- One feels passion towards work.

The scale developed by Dobrow and Tosti-Kharas [10] to measure this calling subtype includes 12 items, and there are versions for musicians, artists and business professionals. The questionnaire captures the following dimensions: An internal source of the call in the form of passion, enjoyment and satisfaction, willingness to sacrifice, identification with the work, a sense that one is destined to perform the work, work meaningfulness and pervasiveness (work being on one’s mind when not working). Items include the following: I am passionate about… (playing my instrument/engaging in my artistic specialty/business/being a manager); The first thing I often think about when I describe myself to others is that I am a…; I feel a sense of destiny about being a… These items capture some but not all the components of the modern calling orientation, as the scale does not include items reflecting a good fit, a sense of self-realization through work, a sense of being called, and seeing work as one’s life purpose, despite their centrality in the conceptual model. Additionally, prosocial goals, and an external source of the call, are also not included in the scale.

Given the centrality of skills, talents and person–job fit in this model, much of the work conducted on this model included occupations in which a special talent or skill is central and essential for conducting the work, such as musicians, artists, businesspeople [10], pilots [145] and military personnel [146].

Similar to the classic calling definitions, the modern conception of calling emphasizes the prominence of callings in people’s lives as a driving force around which their lives are organized, suggesting that work is inseparable from one’s life and strongly tied to one’s identity [16,147]. In contrast to the classic callings, however, modern callings do not involve a prosocial goal, sense of duty or social obligation, or a transcendent summon from an external source [10,19]. In modern callings, the source of the call is internal, reflecting a person’s quest for self-expression and self-actualization of one’s unique interests, skills or talents [20].

Thompson and Bunderson [1] defined this calling subtype as one that is driven by “inner requiredness”, while Dobrow and her colleagues [2] conceptualized this subtype as an “internally driven calling”. Hence, work is performed for its own merit, because of what it means to the person performing it [145], and it entails activities and a vocational path that lead to the fulfilment of one’s unique purpose in life or destiny [10,145,146] through the expression and realization of one’s capacities or skills [10,145,146].

In terms of the location of this calling subtype on the pro-self–prosocial axis, in contrast to the classic subtypes, modern callings are firmly situated on the pro-self side of the pro-self–prosocial axis. It is important to note, however, that it is positioned in the adaptive (as opposed to the maladaptive) part of the axis, and it is therefore low on prosocial goals and behaviors, and high on the pro-self, self-interest-orientated and self-actualizing intents and behaviors [2,5,10].

Examples of pro-self dispositions include perceiving work as “a labor of love”, [145] (p. 928) or a “a lifelong passion” [10] (p. 1002), often sensed as an intense desire or urgency to engage with the work [10,148,149]. Accordingly, this calling orientation has been associated with intrinsic motivation, work engagement and work passion [10,147,150,151].

Contrary to the classic calling subtypes, in the modern calling model, work is tightly linked to one’s talents or skills, and these abilities are seen as the foundation of one’s work and calling [145,148], and therefore, there is a strong person–job fit embedded in this outlook [145,149,152,153]. The fit with the job is often sensed as being highly competent in the occupational domain [145,151,154] or having an “enhanced self-perceived ability in the calling domain” [153] (p. 4), high self-efficacy [145,150,155] or “domain-specific self-esteem” [149] (p. 2). Dobrow [148] noted that having high competence enables the person to experience flow, which necessitates having a match between the requirement of the task that one performs and the person’s skills [156].

Importantly, work is viewed as central to one’s life and identity: “identity is a critical aspect of the calling itself”, and “personal and work identities are tightly intertwined” [148] (p. b3). Work is perceived as one’s purpose in life [10,124] and as an occupation that one was destined to pursue [145,146]. Hence, those holding this outlook “could not imagine doing anything else” [10] (p. 1002).

Dobrow and Tosti-Kharas [10] also found that the calling was perceived as an all-consuming endeavor, whereby the work “engrosses you and encompasses you“, and “totally envelops your life” [10] (p. 1002), hence experiencing what Dobrow [148] (p. b3) described as engulfed consciousness or pervasiveness [10]. The engagement and involvement with the work generate intense meaningfulness both in life and in the work domain [10,147,148], as well as work enjoyment, job satisfaction and pleasure [10,145,147,148].

Michaelson and Tosti-Kharas [6] noted that although this calling outlook is distinctly self-oriented and revolves around a person’s quest for self-realization, the associated dispositions and behaviors were not strictly egoistic. “Rather, self-oriented callings may be ethical in the sense that they enable persons to be their best selves… in the sense of reaching for and realizing one’s own essential identity or ideal character” [6] (p. 27).

These points align well with the literature on self-interest cited earlier, demonstrating the inclination to focus on one’s own needs, desires and interests [60], the tendency to show individualism and independence in one’s pursuits [64], and to follow one’s self-interest goals, or activities that lead to self-expression [60,65]. These points also link with the literature on self-interest in terms of showing achievement motivation [66], self-efficacy or a belief in personal uniqueness [65], inclination to hold an insider perspective in one’s self-perception, manifested in dwelling on their own internal experiences [73], a focus on hedonistic pursuits [65] and cognitive conservatism [73].

The key outcomes of holding this outlook and pursuing this type of calling, beyond the pro-self aspects reviewed above, include enhanced wellbeing [154], life satisfaction [10,151], and optimism [10,151]. However, in another study [157], the authors reported that calling and satisfaction with life were not correlated. Modern calling was also associated with having social capital [146] and experiencing social comfort [151].

In the work domain, a modern calling outlook was positively associated with pursuing higher education [151], long-term participation in the work domain and knowledge of the domain [10], high job involvement [10,147,150,151] and proactive professional development—“a self-driven engagement in work and profession-related learning and developmental activities” [152] (p. 263).

This calling outlook was also associated with various dimensions of career progression and success: career interests, career goals [155], career clarity, clarity of professional identity [10], knowledge of the calling domain [151], career self-efficacy, career confidence and insight, career commitment [10,147,150] and organizational commitment [146]. It was also negatively correlated with career plateaus [146]. However, clarity of professional identity seems to diminish overtime [10].

In terms of performance, since this calling outlook is associated with having a good fit with the work, high performance can be expected, though this is rarely examined in the literature. In several studies, abilities in the calling domain [151,153] were measured among musicians as audition ratings or awards gained in the line of work, which can also be seen as measures of performance; however, these were not significantly correlated with the calling outlook [151,157]. Nevertheless, as noted above, the calling outlook was positively correlated with perceived ability or felt competence [151,154], as well as with self-efficacy [147,150,155]. Interestingly, however, the association between calling and perceived ability was not consistent: Lysova et al. [152] found that the calling outlook was not linked to perceived occupational expertise.

In exploring the costs and sacrifices of this calling subtype, the authors reported that it was associated with overwork [154,158] and an inability to detach from work [154]. Lysova et al. [152] reported on career inflexibility, which Dobrow and Tosti-Kharas [150] described as a “tunnel vision” of one’s career and disregarding well-meant career advice and guidance [150]. The authors [150] also found that musicians with a sense of calling were less realistic in their perception of their own aptitudes, which suggests that they may be pursuing a risky career path [151]. Additionally, counter to expectations, this calling outlook and income were negatively correlated [157].

Thompson and Christiansen [159] argued that the rise of this calling conceptualization concurs with the generational focus on meaningful work. Hence, the process of discovering one’s calling is focused on the needs of the self and the pursuit of fulfilment, passion and enjoyment. Maslow’s [160] conception of self-actualization aligns well with these ideas. Self-actualization is defined as a stage of personal development whereby a person is able to satisfy their needs, express themselves, follow their interests and goals and realize their abilities. In Maslow’s hierarchy of needs, this stage of development concludes in meaningful life and self-fulfillment.

Self-actualization involves [161] (p. 49):

- “The continual actualization and self-expression of potentials, talents and capacities;
- Pursuing valued goals or a mission (or calling, vocation, or a destiny);
- A deeper knowledge of, and acceptance of, one’s intrinsic nature;
- The development of morality and virtues;
- An inclination toward harmony, integration, or synergy within the person”.

Elangovan et al. [9] noted that self-actualizing goals often serve as a motivating force for those pursuing their calling, and Lepisto and Pratt [5] coined the term “realization perspective” to label this calling, which culminates in self-actualization and work enjoyment.

Passion is a central component in modern callings [10]. Perttula and Cardon [162] (p. 192) defined work passion as a “strong inclination toward a self-defining activity that one likes (or loves), finds important, and in which one invests time and energy”. Passion is manifested in the context of modern callings as a strong sense of purpose, and an inner compelling urge to perform the work. Perttula and Cardon [162] considered these manifestations as strong indications of meaningful work. However, Vallerand et al. [163] distinguished between harmonious and obsessive passion, suggesting that in harmonious passion, the desire to engage with the work remains under one’s control, while in obsessive passion, this desire becomes uncontrollable. The authors found that depending on the type of passion, work is likely to result in diligence, enjoyment and commitment, or it may result in a rigid, stressed and obsessed form of engagement. This suggests that the pursuit of one’s calling can become unhealthy if taken to extreme [150].

The findings related to the outcomes of holding a modern calling outlook therefore suggest that it has significant work and career benefits, alongside some costs and sacrifices. However, we note here that only a few personal benefits have been explored in the literature, and as seen, some contradictory findings have emerged.

## 8. The Neoclassical Perspective of Calling

The neoclassic conception of calling was first introduced by Bunderson and Thompson in their study of the zookeepers’ calling [7], and later, it was defined [1] (p.432) as “a conviction—often felt as a sense of destiny or fit—that a particular domain of work leverages one’s particular gifts and consuming passions in service of a cause or purpose beyond self-interest”.

According Bunderson and Thompson [7] and Hagmaier and Abele [164], this calling subtype includes the following core components:

- One feels called to this line of work;
- A prosocial orientation;
- Work is seen as one’s moral duty;
- Good person–job fit;
- Work is tied with one’s life purpose/identity;
- A sense of destiny;
- One experiences passion and work meaningfulness.

Three scales fit with this this model, though some are a better fit than others. The Multidimensional Calling Measure (MCM) developed by Hagmaier and Abele [164] aligns well with the model. It includes 9 items and has three subscales: identification and person–environment fit (self-realization of potential), transcendent guiding force (inner call and destiny), sense and meaning, and prosocial value-driven behavior (serving the common good). Items include the following: I follow an inner call that guides me on my career path; I am passionate about doing my job; I identify with my work; I am destined to do exactly the job I do; by doing my job I serve the common good. It therefore includes a prosocial element alongside a quest for self-realization, and internal drive to perform the work. The scale has no items that directly measure meaningfulness, an external source of the call or seeing work as one’s life purpose, and these are indirectly assessed.

The Neo-classical Calling Scale developed Bunderson and Thompson [7] has the following 6 items: Being called, sense of destiny, occupational identification, good fit, internal call and passion. Items include the following: The work I do feels like my calling in life; I am definitely the sort of person who fits in my line of work; I was meant to do the work I do. The original scale that was used for exploring calling in zookeepers [7] included 27 items and 5 subscales and included items around occupational identification, moral duty, work meaningfulness, occupational importance, willingness to sacrifice, and perceived organizational duty. Example items include the following: I consider it my sacred duty to do all I can for …; The work that I do makes the world a better place; I have a meaningful job. Although the original questionnaire included a prosocial intent and the experience of meaningful work, these were not incorporated into the final version of the scale [7].

Recently, Vianello et al. [15] presented the Unified Multidimensional Calling Scale (UMCS), which brings together items from other scales. It has 22 items, and encompasses seven subscales: identification, pervasiveness, purposefulness, transcendent summons, prosocial orientation, sacrifice and passion. Items include the following: I have been called by something beyond myself to pursue my current line of study; making a difference for others is my primary motivation in my academic and professional career; my academic and professional career is important to give meaning to my life; what I study is part of who I am; what I study is part of my destiny. The scale does not explicitly include items referring to person–job fit. A shorter version of this scale developed by Gerdel et al. [165] includes one item per subscale.

Several studies that drew on this model reported on the existence of this calling outlook within particular occupational sectors including zookeepers [7], animal shelter employees [166], nature preservation staff [167], counselling psychologists [168], physicians [169,170], church staff [171], female leaders in higher education [172], army staff [173,174] and employees in a commercial company [175]. Several studies included employees from various occupations [164,165,176,177].

The evidence drawn from these studies revealed that akin to classic callings, participants express a strong prosocial intent manifested in a sense of duty to serve society [7,162,164,165,168]. Therefore, they perceive the source of their calling as emerging from an external source—a community, an organization, a social cause, or the needs of others. At the same time, comparable to modern callings, these employees have unique skills that fit with the job that they aim to express and realize in their work, and hence, their motivation is also self-focused and driven by a desire for self-realization and fulfillment [7,164,168,170]. As such, analogous to modern callings, the source of their calling is also internal and often perceived as what one is destined to do due to the fit with one’s unique talents.

Neoclassical callings are therefore situated at the midpoint of the pro-self–prosocial axis, due to tying together motives and behaviors that benefit the greater good, alongside pro-self, self-actualizing goals [7]. Conklin [167] described the essence of this calling model, which they framed as “integrated”, as a state where “duty and desire become one” (p. 306), or where one’s values and passions align. Bott et al. [170] (p. 118) explained this point as follows: “the participants’ rationale for fulfilling their calling through medicine reflected both external sources (drawn to serve others or follow a religious/spiritual pull) and internal origins (fit with skills)”.

These descriptions align with Thompson and Bunderson’s [1] claim that among the three calling subtypes, one type entails a match between an “outer requiredness”, which is a societal benefit that the work is geared to accomplish, and “inner requiredness”, which is a distinct set of interests and skills that the person can express and realize that fit well with the work. As mentioned earlier, the authors suggested the title “Transcendent Calling” for this integrated model. Similarly, Dobrow et al. [2] suggested that there is a higher-order calling outlook that ties together internally and externally driven calling outlooks. Although they did not refer to this higher-order calling subtype as neoclassic calling, we maintain that this description is closely aligned with the core features of neoclassic calling.

Prosociality seems to manifest in this model in varied ways, and can include all levels (micro, meso, macro) of prosociality as described by Penner et al. [25], though in the literature, much of the work focuses on the micro (intrapersonal) and meso (interpersonal) levels.

At the micro level, prosocial dispositions documented in the literature include “prosocial motivation to serve others” [170] (p. 118), a commitment to contribute to or benefit others [7], “wanting to make a positive impact” [166] (p. 604), holding “altruistic motives” [178] (p. 54), or an “altruistic focus” [179] (p. 5) or a having a “concern for something beyond one’s own self-interest” [167] (p. 306).

On a meso, interpersonal level [25], prosocial behaviors mentioned in the literature include “serving the greater good or helping other people” [178] (p. 55), reducing suffering [168], acting as “helper, savior, or comforter to others” [169] (p. 153), “being able to contribute effectively to a team and benefit somebody” [178] (p. 55) or having a positive impact on other people [178]. However, the available literature provides little indication as to whether these are voluntary or role-prescribed behaviors.

Alongside these prosocial dispositions and behaviors, several key pro-self dispositions and behaviors feature in the literature. The one most recurrently mentioned was having a particular talent or skill that fits with the work. Bunderson and Thompson [7] (p. 36) reported that for zookeepers, the calling “was grounded in the belief that their basic nature, their hardwiring … predisposed them for a career working with animals” [7] (p. 36). Duffy et al. [168] (p. 302) found that for counselling psychologists “work was a good fit for their skills, values, and interests”. Similar findings were reported by Bott et al. [170] and Nath [169] on medical doctors. Hence, having confidence and self-efficacy in one’s capacities was also an important factor [167,172], culminating in “having greater self-worth” [178] (p. 55).

Love and passion [7,168] were also recurrent themes in the research on this calling subtype that manifested as an intense desire to do “what you love” [167] (p. 304), intrinsic motivation [154], “a strong emotional passion” or “deep passion” [179] (p. 6), following “a path with heart” [167] (p. 305), or being “completely captured” [179] (p. 6) and experiencing an “inexplicable need to pursue” this line of work [178] (p. 55), being proactive, determined and tenacious [179] (p. 7), and in terms of career choice, “never considering anything else” [169] (p. 151).

This calling outlook was also associated with sensing a strong identification with work, in particular with one’s vocational identity [7,168]: “a life calling is who you are, becoming what you do” [179] (p. 4). Longman et al. [172] (p. 266) explained this point: “knowing and using unique giftedness” are “central to the participants’ understanding of who they were.” Another type of alignment reported in the literature is the capacity to pursue one’s life purpose through work [7,164,168], hence experiencing a sense of purpose and direction in life [172,178], meaning in life [170] or work meaningfulness [7,164,172], and feeling “blessed and fortunate to be able to pursue a calling” [178] (p. 56). These culminated in several “internal rewards from the calling” [178] (p. 56): happiness, enjoyment and fulfillment [168,178], career or job satisfaction [164,165,168,177], sense of fulfillment [165,168,178] or feeling rewarded [170].

In line with the modern calling orientation, these points correspond well with the literature on self-interest, demonstrating the centrality of one’s desires and interests [60], the inclination to pursue a one’s self-interest goals or engage with activities that enable self-expression [60], being driven by achievement motivation [66], and having a strong sense of self-efficacy or personal uniqueness [65] and cognitive conservatism [73].

There were several points in which prosocial and pro-self features seem to merge. Bunderson and Thompson [7] maintained that the sense of destiny that features in the neoclassical calling orientation emerges both due to its prosocial component—the contribution of the work that one performs to the greater good—as well as due to having a perfect fit between the job and employee’s interests and talents. Hence, zookeepers felt that their occupation, which centers on animal conservation, is “what one was meant to do” [7] (p. 36), due to having a unique ability to perform it [7] (p. 36). Similar findings emerged in Bott et al.’s [170] (p. 118) study of physicians who noted that “making a difference really drives everything that I do”, and at the same time, they emphasized that a medical career fits their interests, beliefs, skills and passions, and therefore, they felt that it is “what they are meant to do” (p. 122). This integration was manifested in a sense congruence described as “living a life of integrity” [167] (p. 306), “where money doesn’t matter and the prestige doesn’t matter, but more of the value that you find in it” [178] (p. 55), and feeling that “they were on the right track” [178] (p. 55).

The second theme that features in the literature and ties together prosocial and pro-self dispositions is the sense of civic obligation, social responsibility or moral duty that people holding this calling outlook experience [167,169]. However, in contrast to the classic callings, this sense of duty was entwined with the sense of fit with the job [170], and seen as a “moral duty to leverage one’s unique gifts and passions to help humankind” [7] (p. 41) and that “we have been given this gift we are obligated to do something with it for the common good” [167] (p. 305). In some studies, the sense of social responsibility was tied to urgency [167].

The third point that integrates prosocial and pro-self perspective is the source of the call. Similar to the classic calling outlooks, the perception of being summoned to perform the work by an external transcendent source features recurrently in the literature and manifests as being “guided by an external source” [170] (p. 117), “a higher power” [168] (p. 301), or experiencing “a strong, inexplicable force beyond the rational thinking process which led them to pursue their particular career” [178] (p. 55). Hence, they felt “compelled to engage in that call” [167] (p. 306). The external source of the call emanates from a societal need, ranging from a need of a particular client group to a global-scale need [7,166,167,168,170]. At the same time, the literature reports on the existence of an intense internal source of the call to conduct the work, driven by a desire to express and realize one’s talents and skills. For example, Nath’s [169] report on medical doctors makes the following point: the “calling orientation might only develop after the self-identification of proficiency within a particular domain” [169] (p. 154). Schabram and Maitlis [166] (p. 611) reported that animal shelter workers aspired to contribute to a “worthwhile cause” and believed that “they could do this through their distinctive professional skills”. Ahn et al. [178] (p. 54) reported that finding one’s calling occurred alongside the realization of “what I was good at and what I loved to do”. Therefore, this calling orientation is also “about understanding and answering that call from inside of yourself about what you are supposed to do” [178] (p. 57).

Beyond the prosocial and pro-self aspects reviewed above, several personal and work outcomes feature in the literature referring to this calling subtype. Calling was associated with self-awareness [172], psychological capital (hope, self-efficacy, resilience and optimism) [173] and with gratitude [168,170,178]. It was also found to be positively correlated with increased wellbeing [178] and life satisfaction [165,176], though in another study, the association with life satisfaction was not significant [173]. Regarding the sense of significance, interestingly, Bunderson and Thompson [7] (p. 39) reported that “through their identification with the occupation, zookeepers derive a conviction of the significance of their work in society” [7], while Conklin [167] (p. 306) noted that contributing to the greater good resulted in “a reduction in one’s own importance” and “the feeling of insignificance” [167] (p. 306).

Choi et al. [173] found that the calling outlook was positively associated with work-to-family enrichment. A positive association was also found between this calling orientation and social relationships: participants reported having positive interactions with others, which included colleagues, clients, and family members [168,170], and in several studies [7,167], the authors reported on a “convergence in their network of friendships where many of these relationships now are born from their work contacts” (p. 304). Bunderson and Thompson [7] reported on an association between the calling orientation and one’s social affiliation and social identities.

In the work domain, this calling subtype was associated with engagement orientation (tendency to experience flow) [176], performance [171,174], productivity [168,175], sense of career importance [7], organizational identification [7], ideological contract fulfillment [171], career commitment [7,170] and organizational commitment [7,171]. It was also negatively correlated with job demand [175], burnout (work disengagement and exhaustion) [164,176,177] and with an intention to leave the organization [165]. Social support and professional development were found to help sustain the calling over time [170]. Leader’s calling was positively associated with followers’ performance, commitment and attitude, which increases follower perceptions of transformational leadership [174].

Another persistent finding that emerged in several studies [7,166,170] revealed is that the neoclassic calling outlook seems to foster a willingness to make personal sacrifices (such as time, money, comfort and social life) for one’s work. Among physicians [170] (p. 121) a sense of “work-creep” was common, and work was perceived as “labor intensive”, “time-consuming” and “challenging” [170] (p. 118), commonly resulting in an inability to disconnect from work, thus sacrificing down time or family time for work. However, the authors noted that doctors holding this work orientation voiced a sense of obligation to exceed what is in the normative physician description. A similar point was made Bunderson and Thompson [7] (p. 42), wherein zookeepers were continuously “on call” and often asked to come to work outside of regular work hours, and by Schabram and Maitlis [166] (p. 612), who noted “how overworked every position was”. Ahn et al. [178] reported that in order to pursue their calling, people were willing to take a pay cut, and a similar point was made by Bunderson and Thompson [7] (p. 42): “monetary sacrifices are part of the price they pay to be a zookeeper”.

Other sacrifices include engaging with work that is “physically demanding, dangerous, and uncomfortable” [7] (p. 42), working in difficult work conditions [166] such as scarce resources, equipment or lack of training or support [166], experiencing vicarious traumatization [166] and going home “absolutely exhausted” [7] (p. 42). In the social domain, several authors reported on a “lack of social support” around the pursuit of their calling [178] (p. 56), social isolation [179] and that relationships with family and friends were limited, strained or negatively affected [7,170]. In several studies [7,166] the calling was associated with high expectations from themselves and from others, which negatively impacted work relationships and led to criticism and cynicism towards the organization, its leadership and its actions. Bunderson and Thompson [7] voiced a concern that people with a neoclassical calling outlook are at risk of exploitation by employers due to their commitment to work, sense of duty and willingness to make sacrifices [7]. Bott et al. [170] (p. 119) reported that the majority of medical doctors found it challenging to “fulfill their calling through their medical work” due to healthcare limitations, lack of time, and cumbersome administrative work. Schabram and Maitlis [166] reported on some employees experiencing shock, anger, depression, stress, frustration, sadness, discouragement, anxiety and burnout. Given the multitude of sacrifices made by those pursuing their calling, Bunderson and Thompson [7] (p. 39) noted that this type of calling outlook “can be a painfully double-edged sword”.

These studies suggest that neoclassic callings integrate features of classic calling together with characteristics of modern callings. We maintain that this combination makes this category of calling distinct. What is unique about this calling orientation, a point that has not been acknowledged in the literature, is that it moves away from the binary and contrasting conception of pro-self and prosocial intents and actions, suggesting that these concepts can work well in tandem. When tied together, they create an enhanced calling experience that reflects self-actualizing with self-transcendent intents and actions [1,2]. Indeed, there is strong theoretical and empirical correspondence between self-actualization and self-transcendence, and Maslow’s [160] work suggests that self-actualization, as a developmental stage, is a foundation from which self-transcendence can emerge [173,174]. Both are seen as an embodiment of one’s life purpose and can lead to a fulfilled and meaningful life. We note, however, that in this calling outlook, the manifestation of self-transcendent intents and behaviors was less distinct and less central compared to the classic calling orientation.

## 9. Comparative Analysis

### 9.1. Definitions and Components

The analysis presented earlier of the features of classic, modern and neoclassical callings captures the ways in which these constructs are discussed and operationalized in the literature. It reveals that classic, modern and neoclassic callings share several core components:

- The person perceives work as a calling;
- They feel “summoned” to perform the work;
- Work is seen as one’s life purpose;
- Work is seen as a central part of a person’s identity;
- The person experiences work as intensely meaningful.

Drawing on these features, we offer the following overarching definition of calling outlook:

*A work outlook that represents the function and significance of work in a person’s life. The person feels called to perform the work; work is seen as one’s life purpose; it is a central part of the person’s identity; and it renders the work highly meaningful.*

We propose that this construct should be applied in research projects that involve individuals who hold a calling outlook but are not pursuing it [75]. We suggest that a measure should be developed to assess these components, since the existing measures (such as the Brief Calling Scale [14]) do not capture all the components of this overarching definition.

Our analysis also suggests that there are several key points where the three categories of calling diverge.

- Classic calling:
  In addition to the common components of a calling outlook, the secular and religious calling subtypes entail the following:
  - An external source of the call (God, community, organization, others’ needs);
  - A prosocial orientation;
  - A sense of duty to perform the work.

- Modern:
  The distinctive components of a modern calling outlook include the following:
  - The call to conduct the work emerges from within;
  - Good person–job fit;
  - One feels passion towards work;
  - A sense of destiny.

- Neoclassic calling:
  Akin to the other calling subtypes, neoclassic calling entails unique components in addition to the common components cited above:
  - The source of the call is both internal and external;
  - A prosocial orientation;
  - Work is seen as one’s moral duty;
  - Good person–job fit;
  - A sense of destiny;

Next, we examine how these components are measured using the existing scales.

### 9.2. The Measures of Calling

In the comparative analysis, several scales were reviewed: The Faith at Work questionnaire [85] for religious calling, the Calling and Vocation Questionnaire [14] for classic-secular callings, the Modern Calling Scale [10] for modern calling, the Multidimensional Calling scale [164], Neo-classical Calling scale [7], and the Unified Multidimensional Calling Scale (UMCS) [15] and its short version [165] for measuring the neoclassic calling subtype. Table 1 provides a comparison between the components of the scales.

As can be seen from this analysis, the majority of the measures include items that capture:

- A sense of being called to perform the work;
- The alignment of work with one’s life purpose;
- Sense of meaningfulness.

Each of the questionnaires also includes items that measure the unique features of its corresponding calling subtype.

- Classic calling:
  In addition to the above items, the secular and religious calling measures commonly entail:
  - An external source of the call;
  - A prosocial orientation.

- Modern:
  The distinctive questionnaire items of a modern calling outlook include:
  - The call to conduct the work emerges from within;
  - A sense of destiny;
  - Passion/enjoyment;
  - Willingness to sacrifice;
  - Work pervasiveness.

- Neoclassic calling:
  The neoclassic calling scales have in common the following items:
  - The source of the call is internal, external or both;
  - A prosocial orientation;
  - Identification with the work;
  - A sense of destiny;
  - Passion/enjoyment.

As seen in the table, the existing scales do not fully capture the components of their corresponding models (reviewed in Section 9.1) suggesting that there are some construct validity flaws in these scales. The analysis also shows the centrality of the pro-self/prosocial orientation and the source of the call in distinguishing between the calling subtypes. The other items measured are less useful in distinguishing between the calling subtypes. This suggests that there are some convergence validity weaknesses in these scales, which may explain the similarities found in the personal and work-related outcomes that people experience [1,2,4], a point that we will examine below in more depth.

### 9.3. The Pro-Self–Prosocial Continuum

- Positioning on the pro-self–prosocial axis: In the comparative analysis, it emerged that classic religious and secular callings are situated firmly on the prosocial side of the pro-self–prosocial continuum, due to their emphasis on intents and actions that benefit others [14,83]. In contrast, modern calling is situated on the pro-self side of the pro-self–prosocial continuum due to their self-enhancing and self-actualizing goals and behaviors [10], though we note it is located in the adaptive part of the continuum. Neoclassical calling can be situated at the midpoint of the pro-self–prosocial axis, therefore integrating prosocial motives and behaviors with pro-self intents and actions [7].
- Source of the call: In accordance with the positioning of the calling subtypes on the pro-self–prosocial continuum, the source of the calling varies. In classic calling, the source of the calling is external: in a religious calling, one feels summoned to perform the work by a divine source [84], while in a secular calling, the source can be an organization or a community, a social cause or a need [11]. In a modern calling, the source of the call is internal and emanates from one’s desire for self-expression [10]. Neoclassic callings seem to combine an external secular source, alongside an internal source [7].
- Manifestations of pro-self and prosocial dispositions and behaviors: Our analysis suggests that classic and neoclassic calling outlooks can theoretically manifest on all levels of prosociality—micro, meso and macro [25]—involving dispositions and a vast range of behaviors that are geared to benefit others. However, much of the research focuses on the micro (intrapersonal) or on the meso (interpersonal) levels, and the literature is scarce on the macro level. Additionally, in all calling subtypes that involve a prosocial element, the voluntary nature of these prosocial actions needs to be further examined due to some being role prescribed.

In contrast to these manifestations of prosociality, our analysis demonstrates and unpacks the varied pro-self dispositions and behaviors that seem to be at the center of the modern and neoclassic calling models [7,10]. Interestingly, our analysis detected other nuanced differences in the types of prosocial/pro-self dispositions or behaviors displayed across the different subtypes. Firstly, the religious calling subtype shows clearer altruistic (selfless) outlooks and behaviors [83,84] compared to other calling subtypes, though this could be due to research not exploring this point. Another point of divergence is around self-transcendence. The analysis also suggests that self-transcendence manifests more strongly in the two classic calling subtypes compared to neoclassic calling. This is because by definition, self-transcendence requires one to serve a purpose greater than the self with a prosocial intent, and being able to move beyond one’s personal, self-centered ego and needs [99,100]. However, as seen in our analysis, in neoclassic calling pro-self interests and behaviors are a prominent component of this calling orientation, resulting in a less distinct indications of self-transcendence.
A third point refers to the integration of pro-self and prosocial components in the neoclassic calling orientation. While the traditional view considers prosocial and pro-self orientations as two opposing sides of the same axis [100], our analysis suggests that they can be strongly intertwined. This integration becomes evident in the sense of being called deriving both from an internal and an external source [7,166,170,178], one’s sense of being pre-destined to perform the work due to having particular talents or skills that fit with the work [7,167,170,178] and their senses of moral duty to make use of their unique skills in the service of others [7,167,169,170].

The comparative analysis presented here suggests that the positioning of the different calling subtypes on the pro-self–prosocial axis, the source of the call, and the manifestations of pro-self and prosocial dispositions and behaviors seem to be the key factors that differentiate between the three calling subtypes.

### 9.4. Vocational Routes

There is significant evidence to suggest that people in different occupations may perceive their work as a calling [1,2]. However, our analysis suggests that religious callings occur mainly in faith-related occupations [83,84,91,92], classic secular and neoclassic callings are more likely to emerge in occupations that have a strong element of prosociality [7,121,166,168,170], while modern callings are more likely to occur in occupations that revolve around one’s talents or necessitate unique capacities [10,148,152,157].

### 9.5. Outcomes of Calling

Beyond the prosocial and pro-self aspects that are associated with holding this calling outlook, several personal and work outcomes have been mentioned in the literature.

- Personal outcomes: All calling subtypes were positively associated with some indicators of physical or psychological wellbeing, such as life satisfaction [10,130,135,151,164,176], psychological wellbeing [154,178], happiness [134], improved health and health satisfaction [12] and sense of meaning, significance or purpose in life [7,10,83,85,135,137,147,170].

We note, however, that there is limited research on the personal outcomes of calling that enables comparisons between the three subtypes, and we propose that more research is required on aspects that may differ across the calling categories, such as self-actualization and self-transcendence, motivations, prosocial dispositions and behaviors, and values.

- Work outcomes: All categories of callings were positively associated with occupational wellbeing indicators such as job satisfaction [7,10,88,106,135,140,148], sense of work meaningfulness [7,10,106,132,142,164,168,178], identification with the work [83,143,148,168] and intrinsic or extrinsic motivation [14,147,150,151,154].

We note, however, that love and passion [7,10,134,179], work engagement or involvement [7,10,134,150,151,160], work pervasiveness [10,15,145,148], job satisfaction and enjoyment [10,145,147,164,165,168,178], perceived ability, self-esteem and self-efficacy [145,149,150,151,154,155] and the tendency to experience flow [148,176] featured more strongly in modern and neoclassic callings compared to other calling subtypes. This could be, however, because these aspects have been less extensively studied in other calling orientations.
An intriguing question is whether employees with different calling outlooks vary in their performance markers. The literature suggests that secular, modern and neoclassic calling outlooks are positively correlated with performance [119,124,171,175], productivity [168,175], effort [117], effectiveness [117], willingness to handle work challenges [119,121], work ethic and lower absenteeism [133]. However, in one study [107], a religious calling outlook was negatively correlated with performance; in another study, a modern calling outlook was not significantly correlated with performance [151,153]; and a third study [152] reported that a modern calling outlook was not linked to perceived occupational expertise.
Career aspects feature strongly in the calling literature, and researchers reported on a positive association between a calling outlook and varied career success measures such as career importance [7], career commitment [7,10,119,140,147,149,170], career self-efficacy [10,14,130,137,147,150,155] and career adaptability [132].
In terms of one’s relationship with their organization, several studies reported on positive outlooks including organizational loyalty [103], identification with the organization [7,99,143], organizational commitment [7,91,106,140,151,171] and low turnover intentions [7,140,143,165]. However, in one paper [107], the authors reported that a religious calling outlook was positively associated with intents to leave.
The findings suggest that some positive work-related outcomes occur across all calling outlooks, while others may be unique to a particular calling category. However, since there is currently no scale that enables a comparison between classic, modern and neoclassic calling, such differences are not easily detected. We propose that more research is required on work outcomes that may be distinct to each category of calling, such as person–job fit, demands and resources, prosocial organizational behaviors, motivation, work values, occupational and social identities and organizational outcomes.

- Costs and sacrifices: All calling subtypes seem to be associated with willingness to make personal sacrifices for work, such as time, money, comfort and social or family life [7,94,120,130,166,170]. High work demands and overwork was reported across all calling subtypes [93,95,120,125,151,154,157,158,165], often accompanied by an inability to disconnect from work [125,134,154]. This culminated in several mental health issues including depression [125,166], exhaustion [7,119,125] and burnout [108,109,125,166].

Another sacrifice that is typical for those with a religious calling is staying in a job that lacks fit with their skills or is a negative environment [106]. Those holding a neoclassical callings outlook reported lacking work resources training or social support [166,170,178], experiencing social isolation [179] negative work relationships [7], and being at risk of exploitation [7]. Modern callings were also associated with unrealistic self-perceptions and career expectations, career rigidity [150,151,152], ignoring career advice and guidance [150] and pursuing a risky career paths [151]. It was also negatively correlated with income [157].
The comparative account suggests that personal costs and sacrifices due to one’s commitment to the work seem to occur across all calling subtypes. In classic and neoclassic callings, these can be seen as prosocial sacrifices that one is willing to make in order to benefit others. However, in modern callings, they can be framed as costs associated with focusing on and pursuing one’s self-interest.

### 9.6. Refining the Calling Subtypes Definitions

The main distinction that emerges from the comparative analysis, is that classic, modern and neoclassic callings significantly differ in the core values that underlie them along the pro-self–prosocial continuum: classic callings are prosocial and culminate in self-transcendence, modern callings are geared to achieve self-actualization, and neoclassic callings seem to integrate these value systems. Yalom [180] argued that self-transcendent intents and actions involve going beyond the self, and transcending one’s self-interests in order to serve the greater good. The values that underlie self-transcendence are benevolence and universalization [181]. In contrast, self-actualization goals and actions are self-directed and geared towards fulfilling one’s self-interests. They involve a desire for self-expression and achievement. The core values that underlie self-actualization are individualism, self-enhancement and hedonism [181]. Yalom [180] and Schwartz [181] viewed these as contrasting values, and suggested that they imbue life with distinctly different motivations, affective states and meaning. However, as we noted, the neoclassic calling concept moves away from this binary conception of self-actualization and self-transcendence to suggest that these concepts complement rather than contradict each other, and when brought together, they create a unique and powerful calling experience [1,2].

Drawing on the comparative analysis presented above, we offer the following definitions of classic, modern and neoclassic callings:

- Classic calling: *A work outlook that represents the function and significance of work in a person’s life. The person feels summoned to perform the work by an external source (divine or secular, organization or community); work contributes to the greater good and is seen as one’s life purpose and moral duty; it is a central part of the person’s identity; and the person experiences a sense of self-transcendence and intense work meaningfulness.*
- Modern calling: *A work outlook that represents the function and significance of work in a person’s life. The person feels internally driven to perform the work due to a good person–job fit and a desire to achieve self-actualization; work is a central part of the person’s identity and life purpose; and the person experiences passion and enjoyment and an intense work meaningfulness.*
- Neoclassic calling: *A work outlook that represents the function and significance of work in a person’s life. The person feels summoned to perform the work by an external source (divine or secular, organization or community) as well as internally, due to a good person–job fit and a desire to express one’s talents. The work contributes to the greater good and is seen as one’s life purpose and moral duty. It is a central part of the person’s identity, and the person experiences a sense of self-transcendence and self-actualization, and intense work meaningfulness.*

We maintain that these divergent value systems that underpin the different calling subtypes, have been the main source of the disagreement between scholars around the definition of a calling outlook and its components, as they have not been examined empirically nor explored theoretically.

Our analysis regarding the values that underlie the different calling outlooks is aligned with the findings of the taxometric analyses conducted by Hirschi [182] and Shimizu et al. [19]. Hirschi’s [182] analysis identified three calling outlooks: a prosocial calling outlook, which is akin to our definition of classic calling; a career self-centered outlook, in which the key driver is the self-enhancement, which aligns with the modern calling definition; and a third outlook, which the authors described as a positive varied work orientation, whose work values were less distinct, and therefore, it is unclear if they parallel our description of neo-classic callings. Shimizu et al.’s [19] analysis identified two calling sub-categories: one that is akin to classic callings, which the authors described as “pro-social focused”, and a second category that aligns with modern callings, i.e., “self-enhancement focused”. Our analysis also aligns with Thompson and Bunderson’s [1] distinction, cited earlier, between calling subtypes that are driven by “inner requiredness”, “external requiredness” and third type that brings these together. Similarly, our analysis corresponds with Dobrow and et al.’s [2] internally driven and externally driven subtypes, and the higher-order calling subtype, that ties these together.

Our conceptual analysis therefore supplements these empirical analyses by offering a new theoretical lens for analyzing people’s calling experience: the lens of values that prompt and underpin one’s calling and emanate from their positioning on the pro-self–prosocial axis. We argue that this new lens offers a higher level of conceptual clarity in the calling construct compared to existing models, since they identify the foundation of a calling outlook, from which other features of the calling construct emerge. Hence, it can explain where the three calling subtypes converge and diverge.

In contrast, current theoretical models (see, for example, [17]), are mainly geared toward explaining the outcomes that occur across all calling outlooks. While they offer a collective theme, the focus on outcomes masks the underlying value-based inconsistencies and people’s varied experiences of pursuing their calling. Due to this lack of attention to the values that underlie a calling orientation and to the prosocial/pro-self element, these models are unable to offer a firm theoretical foundation for the meta-concept of calling.

## 10. Theoretical Contribution, Limitations and Future Directions

This paper makes several theoretical contributions to the literature on calling. Our intent was to make clearer distinctions between three definitions of calling (classic, modern and neoclassic calling) along a pro-self–prosocial continuum, and thereby advance the scholarship on calling by offering a higher level of conceptual clarity around these key concepts. This was achieved by offering a fine-grained comparative analysis of the three types of calling and by proposing a new overarching definition of a calling outlook, as well as new definitions for each calling subtypes.

The more profound contribution that this paper makes is that it challenges the theoretical models that underpin the current scholarship on calling, which mostly focus on the outcomes of having or living a calling, rather than on the process and experience of holding such outlooks and pursuing one’s calling. We therefore offer an alternative theoretical framework for analyzing people’s calling that centers on the diverse value systems that underpin each category of calling and their respective positioning on the pro-self–prosocial continuum.

Our inquiry suggests that there is a blind spot in the current scholarly work—the absence of systematic examination of the role that pro-self/prosocial values play in callings and how these are linked to the concepts of self-actualization and self-transcendence. We maintain that much of the current conceptual inconsistencies have emerged due to the meager attention to the values that underpin different calling orientations. We suggest that exploring people’s calling experience through the lens of values can resolve these theoretical incongruities by differentiating between calling subtypes that draw on dissimilar value systems.

The practical implication of this paper pertains to the different socialization processes that each calling route necessitates. To encourage young people to find their calling, parents, teachers and counsellors should gain an understanding of the key differences between the calling categories, and support them in embarking on the pathway that is the best fit for them in accordance with their purpose and main motivation.

The main limitation of this paper, particularly in the sections that examine the outcomes of callings, is that the analysis draws on limited empirical (mostly) quantitative literature. This is because our analysis drew from studies that used particular scales which matched the definitions of classic, modern and neoclassic calling subtypes. This led to the exclusion of a significant body of research which has not used these scales.

Drawing on the analysis provided in this paper, future research could develop the value-based theoretical models that underpin different callings. This will deepen our understanding of the function that pro-self/prosocial values play in perceiving or pursuing one’s calling, their impact as drivers of calling, as mechanisms that generate meaning, and their impact on personal or work outcomes. Future research could also revise the existing measures of calling and develop a new scale that can identify classic, modern and neoclassic callings with one scale. Such a measure would enable an empirical comparative analysis between the three sub-categories of calling, to ascertain the difference in their pathways, meanings and outcomes.

## 11. Conclusions

This paper began with the observation that research on calling has been flourishing, but also with an acknowledgement of scholars’ critique that it lacks conceptual clarity. On the back of this critique, we aimed to clarify a central area of confusion and contention—the pro-self/prosocial aspect of a calling outlook: should a calling orientation necessarily entail the pursuit of prosocial goals, or could it be driven by self-focused intents? This point of dispute is reflected in the lack of agreement round the overarching definition of a calling outlook, as well as around the identification of calling subtypes—classic, neo-classic and modern callings [1,4].

Our analysis revealed that classic, modern and neoclassic calling converge around several core features: feeling called, work is one’s life purpose, a part of one’s identity and meaningful. Drawing on these areas of convergence, we offered a new definition of calling as a meta-structure. In addition, the analysis has shown that classic, modern and neoclassic calling diverge along the pro-self-prosocial continuum.

We also noted that these callings are founded on contradictory value systems: classic callings are geared toward self-transcendence, modern callings are directed toward self-actualization, and neoclassic calling incorporates both value systems. Drawing on these distinctions, the paper offers a new definition of each category of calling. We concluded by offering a new theoretical lens for analyzing people’s calling experience: the lens of values on which the calling conception draws.

## Figures and Tables

**Table 1:** Dimensions of classic, modern and neo-classic calling scales.

Scale Reviewed	Classic		Modern	Neo-Classic		
	Religious	Secular				
	Lynn et al. [85].	Dik et al. [14].	Dobrow and Tosti-Kharas [10].	Hagmaier and Abel [164].	Bunderson and Thompson, [7] (both vers).	Vianello et al. [15].
Sense of being called	√	√		√	√	√
External source of the call	√	√			√	√
Internal source of the call			√	√	√	
Prosocial orientation	√	√		√	√	√
Sense of duty/service	√			√	√	
Life purpose	√	√		√	√	√
Identification	√		√	√	√	√
Destiny	√		√	√	√	√
Meaning	√	√	√		√	√
Sacrifice	√		√		√	√
Self-realization				√	√	
Passion/enjoyment/satisfaction			√	√	√	√
Pervasiveness			√			√
